# Comparative Analysis of Short- and Long-Read Sequencing of Vancomycin-Resistant Enterococci for Application to Molecular Epidemiology

**DOI:** 10.3389/fcimb.2022.857801

**Published:** 2022-04-06

**Authors:** Sujin Oh, Soo Kyung Nam, Ho Eun Chang, Kyoung Un Park

**Affiliations:** ^1^ Department of Laboratory Medicine, Seoul National University College of Medicine, Seoul, South Korea; ^2^ Department of Research and Development, PHiCS Institute, Seoul, South Korea; ^3^ Department of Laboratory Medicine, Seoul National University Bundang Hospital, Seongnam, South Korea

**Keywords:** antimicrobial resistance, long-read next-generation sequencing, molecular epidemiology, short-read next-generation sequencing, strain typing, vancomycin-resistant enterococci

## Abstract

Vancomycin-resistant enterococci (VRE) are nosocomial pathogens with genetic plasticity and widespread antimicrobial resistance (AMR). To prevent the spread of VRE in the hospital setting, molecular epidemiological approaches such as pulsed-field gel electrophoresis and multilocus sequence typing have been implemented for pathogen outbreak surveillance. However, due to the insufficient discriminatory power of these methods, whole-genome sequencing (WGS), which enables high-resolution analysis of entire genomic sequences, is being used increasingly. Herein, we performed WGS of VRE using both short-read next-generation sequencing (SR-NGS) and long-read next-generation sequencing (LR-NGS). Since standardized workflows and pipelines for WGS-based bacterial epidemiology are lacking, we established three-step pipelines for SR- and LR-NGS, as a standardized WGS-based approach for strain typing and AMR profiling. For strain typing, we analyzed single-nucleotide polymorphisms (SNPs) of VRE isolates and constructed SNP-based maximum-likelihood phylogenies. The phylogenetic trees constructed using short and long reads showed good correspondence. Still, SR-NGS exhibited higher sensitivity for detecting nucleotide substitutions of bacterial sequences. During AMR profiling, we examined AMR genes and resistance-conferring mutations. We also assessed the concordance between genotypic and phenotypic resistance, which was generally better for LR-NGS than SR-NGS. Further validation of our pipelines based on outbreak cases is necessary to ensure the overall performance of pipelines.

## 1 Introduction

Two enterococcal species, *Enterococcus faecium* and *Enterococcus faecalis*, are frequently associated with human disease outbreaks characterized by high morbidity rates in the context of both hospital- and community-acquired infections (Cetinkaya et al., 2000; Salgado, 2008). Vancomycin-resistant enterococci (VRE) are of particular concern. According to the Antibiotic Resistance Threats Report of the US Centers for Disease Control and Prevention (CDC), approximately 54,500 VRE infections occurred among hospitalized patients in 2017, resulting in an estimated 5,400 estimated deaths and healthcare costs of 539 million dollars (CDC, 2019).

Enterococci have high potential to acquire antimicrobial resistance (AMR) through the transfer of mobile genetic elements or the development of mutations, which constitute advantages over nosocomial pathogens (Arias and Murray, 2012). Glycopeptide antibiotics, such as vancomycin and teicoplanin, are frequently used to treat serious infections caused by multidrug-resistant *Enterococcus* spp. (Munita and Arias, 2016; Ahmed and Baptiste, 2018). However, resistance to glycopeptides, especially vancomycin, has exacerbated the problems caused by enterococcal infections since VRE was first documented in the late 1980s (Heintz et al., 2010). Vancomycin resistance is mediated by various types of *van* gene clusters (*vanA*, *vanB*, *vanC*, *vanD*, *vanE*, *vanG*, *vanL*, *vanM)*, among which *vanA* and *vanB* are the most frequently implicated in VRE outbreaks. *VanA* is responsible for high levels of resistance to vancomycin and teicoplanin, while *vanB* and *vanC* are only associated with vancomycin resistance (Werner et al., 2008; Hollenbeck and Rice, 2012; Munita and Arias, 2016). The transfer of *vanA* and *vanB* occurs through the acquisition of chromosomal transposons between *E. faecium* and *E. faecalis*, while *vanC* is intrinsically present in *Enterococcus gallinarum* and *E. casseliflavus* (Eliopoulos and Gold, 2001). Unfortunately, the situation is worsened by the emergence of resistance to last-resort antibiotics like linezolid and daptomycin which means clinicians often have no viable alternative treatments (Palmer et al., 2011; Klare et al., 2015; Bender et al., 2018).

The widespread AMR of enterococci necessitates strategies for clinical management of infections, including active surveillance and rapid recognition of outbreaks. To elucidate the mechanisms underlying AMR and prevent the dissemination of bacteria, molecular epidemiological analysis of infections is needed. Conventional molecular diagnostic approaches to the analysis of the genetic relatedness of strains include pulsed-field gel electrophoresis (PFGE) and multilocus sequence typing (MLST). PFGE was among the techniques ushering in the era of molecular epidemiology, and has long been considered the gold standard for bacterial strain typing (Goering, 2010; Neoh et al., 2019). However, PFGE is time-consuming, and the choice of restriction enzymes and electrophoresis conditions is limited (Johnson et al., 2007; Pinholt et al., 2015). MLST is another widely used approach in phylogenetic studies, food safety surveillance, and outbreak investigations (Ruiz-Garbajosa et al., 2006). However, MLST has low discriminatory power because it only relies on defining allelic profiles of the seven housekeeping genes (Homan et al., 2002; Carter et al., 2016).

Several recent studies have demonstrated the usefulness of whole-genome sequencing (WGS) for investigating outbreaks. WGS has high discriminatory power for clonal isolates and can reveal the transmission route of hospital infections caused by various species, including methicillin-resistant *Staphylococcus aureus* and carbapenem-resistant Enterobacterales (Madigan et al., 2018; Al Fadhli et al., 2021). Next-generation sequencing (NGS) studies of bacterial pathogens tend to use short-read sequencing, which involves the fragmentation of genomic DNA into short segments of a few hundred nucleotide bases. Rogers et al. (2021) performed a scoping review revealing that Illumina (San Diego, CA, USA) sequencers, which currently dominate the short-read NGS market, are also the most commonly used platforms for sequencing *Enterococcus* spp. Long-read (also known as third-generation) sequencing platforms are designed to yield much longer reads (tens of kilobases) without the need for polymerase chain reaction (PCR) amplification and are currently represented by Single-Molecule Real-time sequencing (SMRT) of Pacific Biosciences (Menlo Park, CA, USA) and Nanopore sequencing (Oxford Nanopore Technologies, Oxford, UK) (Rhoads and Au, 2015). However, their drawbacks include greater sequencing error rates compared to Illumina platforms, although the rates have decreased over recent years (Rang et al, 2018). Especially, the PacBio Sequel system generates assemblies with considerably fewer errors, but the high cost of instruments renders them less accessible (Hrabak et al., 2020; Lang et al., 2020). Nanopore sequencing (Oxford Nanopore Technologies) is cost-efficient and uses portable devices facilitating previously impossible in-field sequencing to be undertaken; thus, this method is suitable for analyzing microbial genomes (Bayley, 2015; Leggett and Clark, 2017).

In this study, we performed bacterial WGS using both short-read and long-read next-generation sequencing (SR-NGS and LR-NGS, respectively) for epidemiological analysis of VRE. We developed pipelines involving three-step analyses of SR- and LR-NGS data for strain typing and AMR profiling, respectively. Moreover, noting the differences between SR- and LR-NGS, the strain typing and AMR detection methods were evaluated using clinical VRE isolates.

## 2 Materials and Methods

### 2.1 Bacterial Isolates

A total of 23 samples, including 13 clinical isolates and ten College of American Pathologist samples were analyzed in this study. Clinical isolates were collected from Seoul National University Bundang Hospital. For each isolate, species identification was performed and AMR was detected using the MicroScan WalkAway system (Beckman Coulter, Brea, CA, USA). Three species were identified as *E. faecalis*; the remainder were *E. faecium*. The AMR of each isolate to vancomycin, teicoplanin, gentamicin, streptomycin, tetracycline, and daptomycin was detected based on the minimal inhibitory concentration (MIC), in accordance with Clinical & Laboratory Standards Institute (CLSI) guidelines (Clinical and Laboratory Standards Institute [CLSI], 2021). For isolates that were phenotypically vancomycin resistant, an additional *van* gene specific PCR test was performed to determine the type of vancomycin-resistant gene (*vanA*, *vanB*, or *vanC*).

### 2.2 Sample Preparation

Prior to WGS of the VRE isolates, each isolate was cultured on a blood agar plate and subjected to DNA extraction. Genomic DNA extraction of colonies was performed using InstaGene Matrix (Bio-Rad Laboratories, Hercules, CA, USA) according to the manufacturer’s instructions and the concentration was measured using a Quantus fluorometer (Promega, Madison, WI, USA).

### 2.3 Library Preparation and Sequencing

#### 2.3.1 Short-Read Next-Generation Sequencing on the iSeq 100 Platform

For SR-NGS, WGS of bacterial DNA was performed using the iSeq 100 (Illumina). DNA libraries were prepared using the Nextera DNA Flex Library Prep Kit (Illumina) according to the manufacturer’s instructions. After adapter ligation, barcoding was performed using the Nextera Index primers (Illumina); 23 isolates per flow cell were run in multiplex. The completed sample libraries were quantified using the Quantus fluorometer to achieve a standardized input of 100 ng per sample. Libraries were pooled and diluted to the final loading concentration of 200 pM with 1% PhiX (Illumina) spike-in, and sequenced on iSeq 100 i1 REAGENT cartridge (Illumina) using 150-bp paired-end reads.

#### 2.3.2 Long-Read Next-Generation Sequencing on the MinION Platform

MinION (Oxford Nanopore Technologies) was chosen as the LR-NGS platform and WGS was performed on 20 isolates (DNA samples of three isolates were not available). For library preparation, DNA was end-repairelid and A-tailed with NEBNext FFPE DNA Repair Mix and the NEBNext Ultra II End Repair/dA-Tailing Module (New England Biolabs, Ipswich, MA, USA). Ligation of the sequencing adapters was performed using the SQK-LSK109 kit (Oxford Nanopore Technologies) and each sample was barcoded using the EXP-NBD104 kit (Oxford Nanopore Technologies). Each step was followed by purification with Agencourt AMPure XP beads (Beckman Coulter). Libraries were pooled in equimolar amounts and adjusted to a final concentration of 1 pM. Twenty isolates were sequenced per flow cell (FLO-MIN106D; Oxford Nanopore Technologies) using MinKNOW software (ver. 20.10; Oxford Nanopore Technologies).

### 2.4 Data Quality Control and Preprocessing

#### 2.4.1 Preprocessing of Short Reads

Demultiplexed FASTQ files were exported from iSeq 100 and subjected to quality control (QC) using FastQC (Babraham Bioinformatics, Cambridge, UK). The QC indices were chosen regarding manufacturer’s recommendations and observed run data. Specifically, we evaluated the iSeq data according to the number and mean length of the reads, percentage of reads with an average quality (Phred) score > 30, and the GC content. Then, the reads were trimmed and filtered using CLC Genomics Workbench (ver. 21.0.4; Qiagen, Hilden, Germany). The quality threshold was 0.05 and the maximum read length was 150 bp.

#### 2.4.2 Preprocessing of Long Reads

Raw fast5 files were basecalled, sorted according to their barcodes, and converted into FASTQ format using Guppy v.4.2.2 (Oxford Nanopore Technologies). Trimmed reads with an average quality score > 7 were retained. EPI2ME, a real-time bioinformatic tool specifically designed for Nanopore sequencing, was used to conduct QC of reads in conjunction with the What’s In My Pot (WIMP) workflow. The QC statistics of the MinION reads included quality scores, sequence lengths, and overall yields. Length filtering was performed using CLC Genomics Workbench; reads shorter than 500 bp were discarded.

### 2.5 Single-Nucleotide Polymorphism-Based Strain Typing

For SR-NGS, downstream analyses were conducted using the Microbial Genomics Module of CLC Genomics Workbench. After acquiring the trimmed FASTQ data, the reads were mapped to the reference sequence of the DO *E. faecium* strain (National Center for Biotechnology Information [NCBI] accession no. NC_017960) using the “map reads to reference” function of CLC Genomics Workbench. On the other hand, the trimmed MinION data were subjected to reference-based assembly using the read mapping function of the Long Read Support plugin, which is specifically used for the assembly of error-prone long reads. Variant calling of mapped reads was performed using the “basic variant detection” function, and genetic alterations including single-nucleotide variants (SNVs) and small insertions and deletions (INDELs) were detected. Variants with coverage below 10 and frequency lower than 35% were discarded. The variant calls of each isolate were combined and the nucleotide positions were determined. The consensus sequence of each isolate was aligned to determine substitutions at specific positions and single-nucleotide polymorphisms (SNPs) were identified. The trees were constructed using maximum-likelihood method under the Jukes–Cantor (Jukes and Cantor, 1969) nucleotide substitution model. Bootstrapping was conducted with 100 replicates.

### 2.6 Antimicrobial Resistance Profiling

AMR profiling involves finding genes and point mutations conferring resistance to antibiotics. In this study, trimmed short reads were subjected to *de novo* assembly prior to AMR analysis. For LR-NGS, an additional error correction process was applied *via* partial order alignment (POA) algorithms using the Long Read Support plugin (Lee et al., 2002). Contigs comprising short reads or corrected long reads were compared against the Comprehensive Antibiotic Resistance Database (CARD) (Alcock et al., 2020), Antibiotic Resistance Gene-ANNOTation (ARG-ANNOT) database (Gupta et al., 2014), ResFinder (Bortolaia et al., 2020), and the NCBI database. The minimum identity between the AMR genes in the database and SR- and LR-NGS sequences was set to 90%. To determine the presence of resistance-conferring mutations of antibiotic target genes, the PointFinder (Zankari et al., 2017) database was used.

### 2.7 Data Visualization and Statistical Analysis

Data visualization and statistical analyses were conducted using R software (ver. 4.1.0; R Development Core Team, Vienna, Austria). The Wilcoxon signed rank test was used to compare two groups of non-parametric distribution. Maximum-likelihood phylogenies of SR- and LR-NGS, generated using CLC Genomics Workbench, were exported into R software in Newick format for further analysis. The *ggtree* R package was used for visualization and annotation of the phylogenetic trees (Yu et al., 2017). For comparison of two phylogenetic trees, the tanglegram algorithm was performed using the *dendextend* R package (Galili, 2015). The tanglegram algorithm compares two phylogenies by placing them next to each other and drawing lines to connect corresponding taxa (identified based on identical tip labels). This algorithm minimizes the number of crossing connectors; identical trees have no crossing connectors (Scornavacca et al., 2011).

## 3 Results

### 3.1 Overview of Sequencing Data

VRE isolates were subjected to SR-NGS and QC was performed using FastQC. The FastQC results indicated that a total of 5,149,064 reads were generated per run, and were of high quality (mean q-score was above 34 in all isolates). The mean read length of each isolate ranged from a minimum of 136 bases to a maximum of 147 bases (**Table 1**). The quality of the sequencing data of the 20 isolates produced by LR-NGS was assessed using EPI2ME based on several indices. The MinION platform generated 1,609,088 reads (1,349 Mbases per run) and provided the longest read length of 51,863 bases. The mean q-score of the reads was a minimum of 8.3 and a maximum of 8.7, and the average length varied from 756 to 940 bases (**Table 2**).

**Table 1:** Quality control results of FastQC for the raw short-read next-generation sequencing data.

Isolate	Reads	Mean length	Mean Q	%Q30	GC%
EF01	193500	138.17	34.22	93.03	36
EF02	67044	144.39	34.33	93.92	37
EF03	531506	142.92	34.11	92.36	36
EF04	273490	145.26	34.06	92.04	36
EF05	91784	141.88	34.23	93.22	36
EF06	73214	141.94	34.32	94.23	36
EF07	440134	146.56	34.11	92.37	35
EF08	302150	146.69	34.09	92.33	36
EF09	123002	144.29	34.23	93.50	35
EF10	79648	143.82	34.30	93.66	36
EF11	180582	145.15	34.33	94.18	36
EF12	23474	141.69	34.36	94.29	36
EF13	140412	138.83	34.37	94.31	33
EF14	53982	135.78	34.19	93.17	36
EF15	34600	139.05	34.43	94.70	36
EF16	420790	143.95	34.10	92.12	35
EF17	239628	140.83	34.20	93.01	36
EF18	244838	142.62	34.37	94.57	50
EF19	566650	141.53	34.18	92.94	36
EF20	433036	137.86	34.28	93.29	36
EF21	281094	140.26	34.01	91.85	44
EF22	119860	142.58	34.37	93.99	38
EF23	234646	146.12	34.00	91.66	42
Minimum	23474	135.78	34.00	91.66	33
Maximum	566650	146.69	34.43	94.70	50
Average	223872	142.27	34.23	93.25	37.09

Q, quality score; %Q30, percentage of reads with a quality score > 30.

**Table 2:** Quality control results of EPI2ME for the raw long-read next-generation sequencing data.

Isolate	Reads	Mean length	Bases	Mean Q	%Q		
					%Q7	%Q9	%Q12
EF01	121786	828.53	100903788	8.42	100.00	22.92	0.27
EF02	47108	838.77	39512611	8.42	100.00	22.10	0.22
EF03	114267	834.69	95377335	8.47	100.00	24.47	0.31
EF04	16015	905.70	14504865	8.38	100.00	21.12	0.18
EF05	18121	821.51	14886591	8.27	100.00	17.55	0.14
EF06	43279	756.26	32729984	8.35	100.00	19.84	0.15
EF07	16391	909.97	14915270	8.52	100.00	27.53	0.30
EF08	43191	939.54	40579505	8.45	100.00	23.76	0.24
EF09	81437	896.46	73004798	8.40	100.00	22.14	0.20
EF10	60704	892.42	54173505	8.34	100.00	19.63	0.17
EF11	125774	910.26	114487045	8.47	100.00	23.98	0.30
EF12	24779	771.36	19113414	8.28	100.00	17.21	0.20
EF13	26114	842.03	21988669	8.72	100.00	33.20	0.83
EF14	44000	767.00	33748184	8.57	100.00	27.31	0.41
EF15	24000	757.86	18188524	8.52	100.00	25.14	0.54
EF16	159372	801.09	127671311	8.67	100.00	31.78	0.67
EF17	52000	841.40	43752949	8.51	100.00	25.60	0.47
EF21	370750	797.24	295576153	8.57	100.00	28.82	0.41
EF22	48000	918.98	44111054	8.30	100.00	16.73	0.22
EF23	172000	871.35	149872845	8.52	100.00	25.73	0.37
Minimum	16015	756.26	14504865	8.27	100.00	16.73	0.14
Maximum	370750	939.54	295576153	8.72	100.00	33.20	0.83
Average	80454	845.12	67454920	8.46	100.00	23.83	0.33

Q, quality score; %Q7, percentage of reads with a quality score > 7; %Q9, percentage of reads with a quality score > 9; %Q12, percentage of reads with a quality score > 12.

### 3.2 Primary Analysis

#### 3.2.1 Data Preprocessing and Read Assembly

Following quality filtering and length trimming, the median ratio of trimmed short reads to raw reads was 99.72, compared to 79.8 for the long reads (data not shown). The preprocessed reads of 20 isolates were assembled using the reference sequence of *E. faecium*, and five isolates had a noticeably lower depth of coverage than the others. Two of those five isolates were *E. faecalis*, consistent with the results obtained by MicroScan WalkAway prior to DNA extraction. However, for the other three isolates, discordance was observed between the sequencing results and MicroScan WalkAway identifications. While all three isolates were confirmed to be *E. faecium* by biochemical tests, *E. faecalis* and *E. casseliflavus* were present in two of them according to the WIMP analysis performed using EPI2ME. The remaining species was identified as *Bacillus* spp. and thus excluded from the downstream analysis.

### 3.3 Secondary Analysis

#### 3.3.1 Comparison of Short- and Long-Read Sequencing: Variant Calling

**Figure 1:**
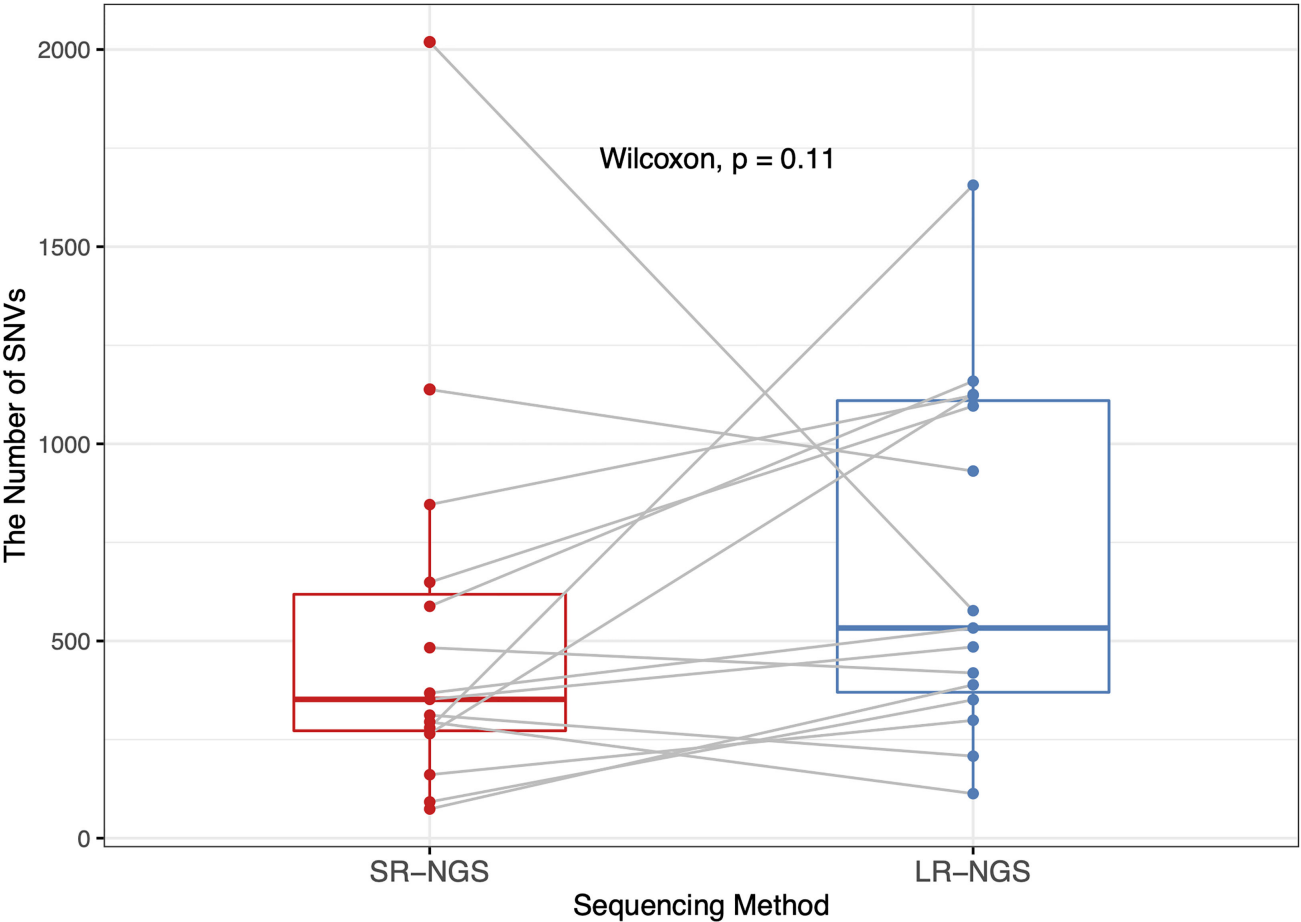
Comparison of the numbers of single-nucleotide variants (SNVs) in vancomycin-resistant *Enterococcus faecium* isolates identified by short-read next-generation sequencing (SR-NGS) and long-read next-generation sequencing (LR-NGS).

Fifteen vancomycin-resistant *E. faecium* (VREfm) isolates mapped to the reference genome were subjected to variant calling using CLC Genomics. For comparative analysis of the SNVs generated by the short- and long-read sequencing platforms, a nonparametric test was performed to assess differences in the number of SNVs; the results are plotted in **Figure 1**. LR-NGS detected more SNVs than SR-NGS, but the difference was not significant (p = 0.11). Meanwhile, the median number of INDELs generated from long reads was significantly higher than that generated from short reads, which reflects the indel dominating errors of Nanopore sequencing (p = 3.105 × ^10−6^; data not shown).

### 3.4 Tertiary Analysis

#### 3.4.1 Comparison of Short- and Long-Read Sequencing: Single-Nucleotide Polymorphism-Based Strain Typing

**Figure 2:**
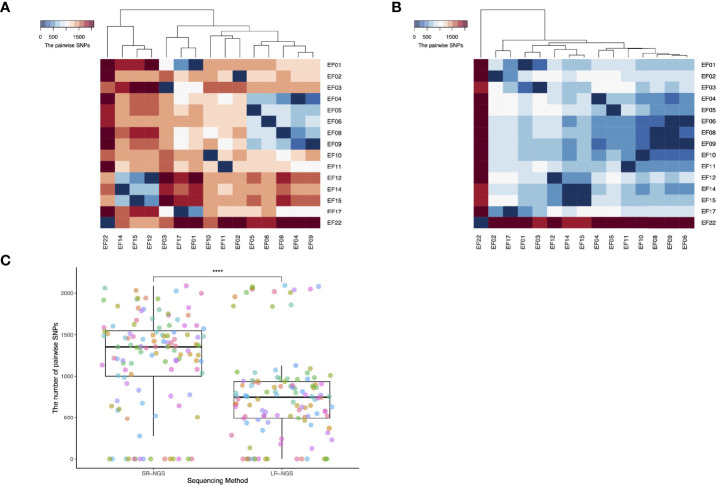
Single-nucleotide polymorphisms (SNPs) detected by whole-genome sequencing of vancomycin-resistant *Enterococcus faecium* (VREfm) isolates. **(A)** and **(B)** show the pairwise SNPs generated by short-read next-generation sequencing (SR-NGS) and long-read next-generation sequencing (LR-NGS). Each row and column in both heatmaps indicates a VREfm isolate, and the columns were clustered using the hierarchical clustering method. **(C)** The Wilcoxon rank-sum test showed that the number of SNPs detected differed significantly between the two methods (****: p-value < 0.0001).

**Figure 3:**
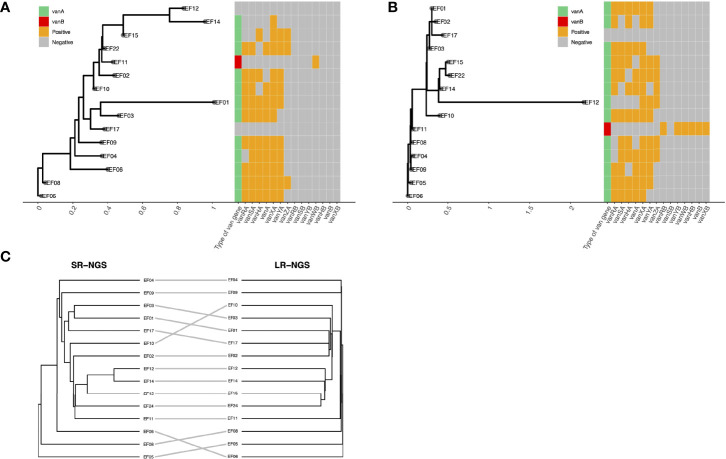
Comparison of the single-nucleotide polymorphism-based strain typing results of short-read next-generation sequencing (SR-NGS) and long-read next-generation sequencing (LR-NGS) for vancomycin-resistant *Enterococcus faecium*. **(A)** and **(B)** depict the maximum-likelihood phylogenies constructed using SR- and LR-NGS, respectively. The heatmaps on the right show the *van* gene clusters. **(C)** shows a tanglegram of the two phylogenetic trees.

The SNPs between the two VREfm isolates were identified and visualized using a heatmap. As shown in **Figures 2A, B**, the two isolates with the most pairwise SNPs were EF22 and EF08, followed by EF22 and EF04, according to both SR- and LR-NGS. However, the overall number of SNPs detected by SR-NGS was greater than that detected by LR-NGS (p < 0.05; **Figure 2C**). Based on the SNPs detected by SR- and LR-NGS, maximum-likelihood phylogenies were constructed using the Jukes–Cantor nucleotide substitution model (**Figures 3A, B**). Tanglegrams were generated for visual comparison of two SNP-based maximum-likelihood phylogenies; the results are shown in **Figure 3C**. The tanglegram algorithm yielded two dendrograms; a few connectors crossed, indicating that the phylogenetic trees produced by SR- and LR-NGS were not identical. Despite the minor differences, the correspondence between the trees was good overall.

#### 3.4.2 Comparison of Short- and Long-Read Sequencing: Bioinformatic Profiling of Antimicrobial Resistance

Various types of AMR (i.e., to vancomycin, teicoplanin, aminoglycoside, tetracycline, macrolide–lincosamide–streptogramin [MLS], and trimethoprim) were evaluated based on the presence of AMR genes and resistance-inducing point mutations in the assembled contigs of short reads and corrected long reads. No point mutation leading to AMR was found, but several AMR genes were detected. For the representative antibiotics, the phenotypic resistance determined by MIC and genotypic resistance predicted by SR- and LR-NGS were compared. The representative antibiotics refer to vancomycin, teicoplanin, aminoglycoside, tetracycline, and daptomycin.

##### 3.4.2.1 Glycopeptide Resistance

**Figure 4:**
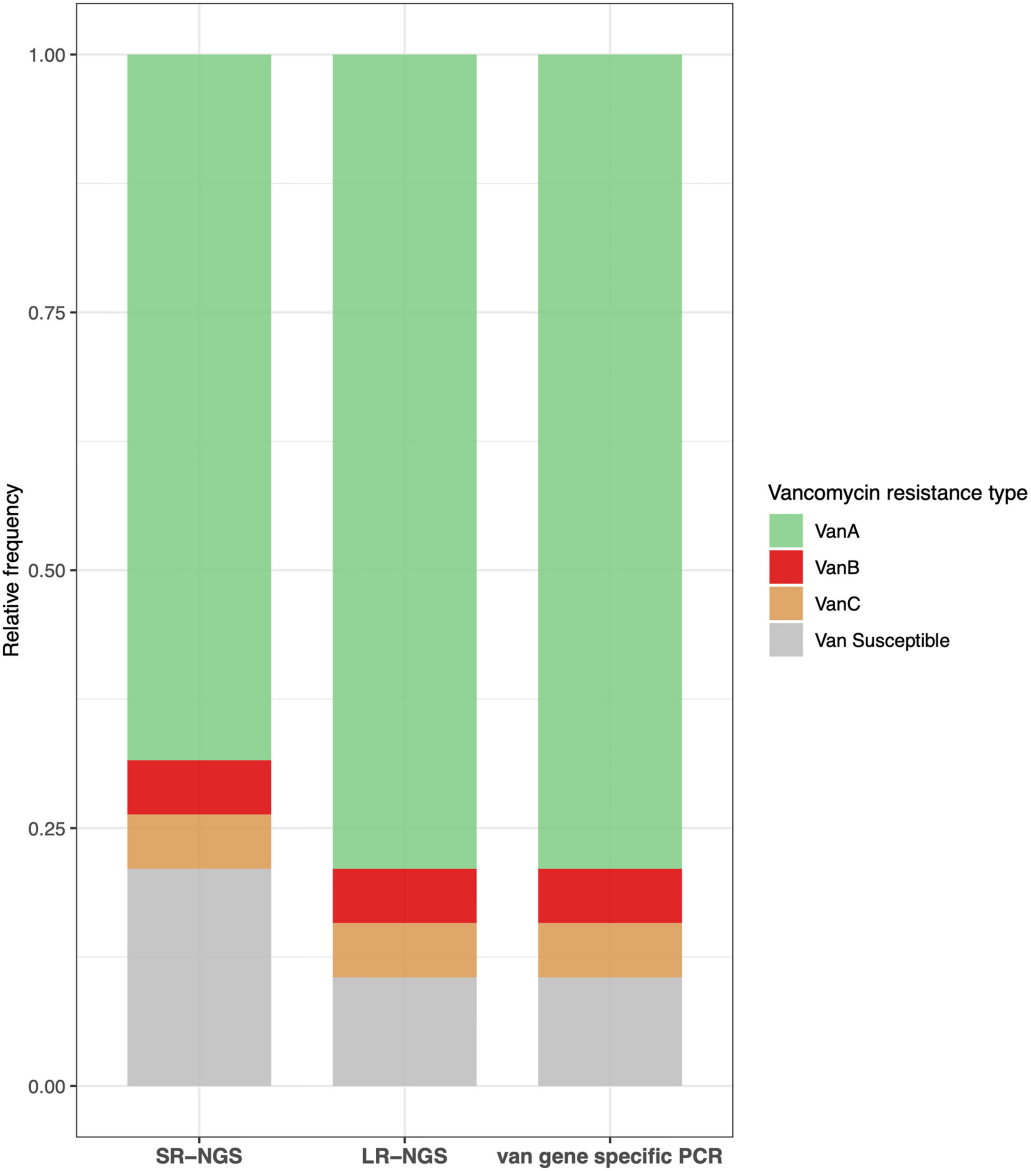
Concordance between the vancomycin resistance results obtained by short-read next-generation sequencing (SR-NGS), long-read next-generation sequencing (LR-NGS), and *van* gene specific polymerase chain reaction (PCR). The types of vancomycin resistance revealed by LR-NGS accorded with those confirmed by *van* gene specific PCR, while few vanA isolates were classified as vancomycin susceptible by SR-NGS.

Vancomycin resistance was determined based on the presence of *van* gene clusters; *vanA*, *vanB*, and *vanC* were the three most prevalent *van* genes in VRE isolates. A *vanA* cluster consisting of seven genes (*vanRSHAXYZ*) was detected in 13 isolates using SR-NGS, and in 15 isolates using LR-NGS. A *vanB* gene cluster consisting of seven genes (*vanRSYWHBX*) and *vanC* cluster consisting of five genes (*vanC(XY)TRS*) were detected in a single sample by both SR- and LR-NGS. The type of vancomycin resistance was confirmed using the MicroScan WalkAway system followed by PCR. The results were compared to those of the NGS vancomycin resistance profiling. Although the vancomycin resistance revealed by LR-NGS accorded with the PCR genotyping results, two *vanA* isolates were classified as vancomycin susceptible by SR-NGS (**Figure 4**). Regardless of the sequencing platform used, the *E. casseliflavus* isolate was found to harbor *vanC* resistance.

Teicoplanin resistance was determined based on the presence of the *vanA* gene cluster and a MIC breakpoint of > 16 μg/mL. Among the nine isolates found to be phenotypically resistant to teicoplanin, seven were *vanA* resistant according to SR-NGS, whereas all nine were *vanA* resistant according to LR-NGS. For both vancomycin and teicoplanin, all the phenotypically resistant isolates were confirmed to be resistant by LR-NGS (**Supplementary Table 1**).

##### 3.4.2.2 Aminoglycoside Resistance

Each isolate was considered to be aminoglycoside resistant if genes encoding aminoglycoside-modifying enzymes (AMEs) were detected. SR- and LR-NGS AMR profiling revealed *AAC(6’)-Ii*, which belongs to the aminoglycoside acetyltransferase (AAC) family of AMEs, in ten and 13 isolates, respectively; *AAC(6’)-Ii* was found in nine of the isolates by both methods. The *aad(6)* aminoglycoside nucleotidyltransferase (ANT) gene, which confers resistance to high-level streptomycin, was found in two isolates by both SR- and LR-NGS, while the *ANT(9)-Ia* gene was found in four and six isolates, respectively. According to both SR- and LR-NGS, six isolates harbored the *APH(3’)-IIIa* gene, which encodes an aminoglycoside phosphotransferase (APH); five of those isolates were common to both methods. Finally, the *AAC(6’)-Ie-APH(2’’)-Ia* gene, which encodes a bifunctional enzyme acting on all aminoglycosides except streptomycin, was detected by SR- and LR-NGS in five and six isolates, respectively; four of those isolates were common to both methods. **Tables 3**, **4** show the isolates harboring aminoglycoside-resistant genes, as detected by SR- and LR-NGS.

**Table 3:** Genes detected by short-read next-generation sequencing that confer antimicrobial resistance (AMR) to aminoglycoside.

Isolate	AMR genes conferring aminoglycoside resistance				
	*AAC(6’)-Ii*	*aad(6)*	*ANT(9)-Ia*	*APH(3’)-IIIa*	*AAC(6’)-Ie-APH(2’’)-Ia*
EF01	**+**	**-**	**-**	**-**	**-**
EF02	**-**	**-**	**-**	**-**	**-**
EF03	**+**	**-**	**+**	**-**	**-**
EF04	**+**	**-**	**+**	**+**	**+**
EF05	**+**	**-**	**-**	**+**	**-**
EF06	**-**	**-**	**-**	**+**	**-**
EF07*	**-**	**-**	**-**	**-**	**+**
EF08	**+**	**+**	**+**	**+**	**-**
EF09	**+**	**-**	**-**	**+**	**+**
EF10	**-**	**-**	**-**	**-**	**-**
EF11	**+**	**-**	**-**	**+**	**+**
EF12	**-**	**-**	**-**	**-**	**-**
EF13*	**-**	**-**	**-**	**-**	**+**
EF14	**+**	**+**	**-**	**-**	**-**
EF15	**-**	**-**	**-**	**-**	**-**
EF16*	**-**	**-**	**-**	**-**	**-**
EF17	**+**	**-**	**+**	**-**	**-**
EF22	**+**	**-**	**-**	**-**	**-**
EF23†	**-**	**-**	**-**	**-**	**-**
**Total**	10	2	4	6	5
	**13**				

*Enterococcus faecalis.

^†^Enterococcus casseliflavus.

**Table 4:** Genes detected by long-read next-generation sequencing that confer antimicrobial resistance (AMR) to aminoglycoside.

Isolate	AMR genes conferring aminoglycoside resistance				
	*AAC(6’)-Ii*	*aad(6)*	*ANT(9)-Ia*	*APH(3’)-IIIa*	*AAC(6’)-Ie-APH(2’’)-Ia*
EF01	**+**	**-**	**+**	**-**	**-**
EF02	**+**	**-**	**+**	**-**	**-**
EF03	**+**	**-**	**+**	**-**	**-**
EF04	**+**	**-**	**-**	**+**	**-**
EF05	**-**	**-**	**-**	**-**	**-**
EF06	**+**	**-**	**+**	**+**	**+**
EF07*	**-**	**-**	**-**	**-**	**+**
EF08	**+**	**+**	**+**	**+**	**-**
EF09	**+**	**-**	**-**	**+**	**+**
EF10	**+**	**-**	**-**	**-**	**+**
EF11	**+**	**-**	**-**	**+**	**+**
EF12	**-**	**-**	**-**	**+**	**-**
EF13*	**-**	**-**	**-**	**-**	**+**
EF14	**+**	**+**	**-**	**+**	**-**
EF15	**+**	**-**	**-**	**-**	**-**
EF16*	**-**	**-**	**-**	**-**	**-**
EF17	**+**	**-**	**+**	**-**	**-**
EF22	**+**	**-**	**-**	**-**	**-**
EF23†	**-**	**-**	**-**	**-**	**-**
**Total**	13	2	6	6	6
	**16**				

*Enterococcus faecalis.

^†^Enterococcus casseliflavus.

Phenotypic resistance of high-level gentamicin and streptomycin was determined by MIC breakpoints of > 500 and > 1,000 μg/mL, respectively. All nine isolates that were phenotypically resistant to gentamicin and streptomycin harbored at least one AME gene according to LR-NGS. However, no AME gene was detected by SR-NGS in one gentamicin-resistant isolate, or in an isolate resistant to both gentamicin and streptomycin. Concordance between phenotypic and genotypic resistance was observed for seven of the nine VRE isolates. Every isolate that showed the phenotypic resistance to gentamicin, streptomycin, or both were also identified as aminoglycoside-resistant strains by LR-NGS (**Supplementary Table 1**).

##### 3.4.2.3 Tetracycline Resistance

Tetracycline resistance was inferred based on the existence of *tet* genes (**Tables 5**, **6**). SR-NGS showed that four isolates harbored the *tetM* gene, which encodes a tetracycline-resistant ribosomal protection protein, while one isolate also harbored the *tetU* gene, which encodes the major facilitator superfamily antibiotic efflux pump. Meanwhile, LR-NGS showed that one isolate harbored the *tetM* gene. None of the above isolates were classified as tetracycline resistant by both sequencing methods.

**Table 5:** Genes detected by short-read next-generation sequencing that confer antimicrobial resistance (AMR) to tetracycline and trimethoprim.

Isolate	AMR genes conferring tetracycline resistance			AMR genes conferring trimethoprim resistance						
	*tetM*	*tetU*		*dfrC*	*dfrE*		*dfrF*		*dfrG*	
EF01	**-**	**-**		**-**	**-**		**-**		**-**	
EF02	**-**	**-**		**-**	**-**		**-**		**-**	
EF03	**+**	**-**		**-**	**-**		**-**		**+**	
EF04	**+**	**-**		**-**	**-**		**-**		**+**	
EF05	**-**	**-**		**-**	**-**		**-**		**-**	
EF06	**-**	**-**		**-**	**-**		**-**		**-**	
EF07*	**-**	**-**		**-**	**-**		**-**		**+**	
EF08	**+**	**+**		**-**	**-**		**-**		**+**	
EF09	**-**	**-**		**-**	**-**		**-**		**-**	
EF10	**-**	**-**		**-**	**-**		**-**		**-**	
EF11	**-**	**-**		**-**	**-**		**-**		**-**	
EF12	**-**	**-**		**-**	**-**		**-**		**-**	
EF13*	**-**	**-**		**-**	**-**		**-**		**-**	
EF14	**-**	**-**		**-**	**-**		**-**		**-**	
EF15	**-**	**-**		**-**	**-**		**-**		**-**	
EF16*	**+**	**-**		**-**	**-**		**-**		**-**	
EF17	**-**	**-**		**-**	**-**		**-**		**-**	
EF22	**-**	**-**		**-**	**-**		**-**		**-**	
EF23†	**-**	**-**		**-**	**-**		**-**		**-**	
**Total**	4		1	0		0		0		4
	**4**			**4**						

*Enterococcus faecalis.

^†^Enterococcus casseliflavus.

**Table 6:** Genes detected by long-read next-generation sequencing that confer antimicrobial resistance (AMR) to tetracycline and trimethoprim.

Isolate	AMR genes conferring tetracycline resistance		AMR genes conferring trimethoprim resistance			
	*tetM*	*tetU*	*dfrC*	*dfrE*	*dfrF*	*dfrG*
EF01	**-**	**-**	**-**	**-**	**-**	**+**
EF02	**-**	**-**	**-**	**-**	**-**	**-**
EF03	**-**	**-**	**-**	**-**	**-**	**+**
EF04	**-**	**-**	**-**	**-**	**-**	**+**
EF05	**-**	**-**	**-**	**-**	**-**	**-**
EF06	**-**	**-**	**-**	**-**	**-**	**+**
EF07*	**-**	**-**	**-**	**+**	**-**	**+**
EF08	**-**	**-**	**-**	**-**	**-**	**+**
EF09	**-**	**-**	**-**	**-**	**-**	**-**
EF10	**-**	**-**	**-**	**-**	**-**	**+**
EF11	**-**	**-**	**-**	**-**	**-**	**-**
EF12	**-**	**-**	**-**	**-**	**-**	**-**
EF13*	**-**	**-**	**+**	**-**	**-**	**-**
EF14	**-**	**-**	**-**	**-**	**+**	**-**
EF15	**-**	**-**	**-**	**-**	**-**	**-**
EF16*	**-**	**-**	**-**	**+**	**-**	**-**
EF17	**-**	**-**	**-**	**-**	**-**	**-**
EF22	**+**	**-**	**-**	**-**	**-**	**-**
EF23†	**-**	**-**	**-**	**-**	**-**	**-**
**Total**	1	0	1	2	1	7
	**1**		**10**			

*Enterococcus faecalis.

^†^Enterococcus casseliflavus.

The phenotypic resistance of tetracycline was determined based on a MIC breakpoint of > 8 μg/mL. Among the five isolates found to be phenotypically resistant to tetracycline, one was confirmed to harbor the *tet* gene according to both SR- and LR-NGS (**Supplementary Table 1**).

##### 3.4.2.4 Trimethoprim Resistance

Trimethoprim resistance was determined based on the presence of *dfr* genes coding for trimethoprim-resistant dihydrofolate reductase. According to SR-NGS, four isolates harbored *dfrG* genes (**Table 5**), while LR-NGS detected *dfrG* genes in seven isolates; *dfrC*, *E*, and *F* genes were also detected by the latter method (**Table 6**). Four isolates were genotypically resistant to trimethoprim according to SR-NGS, compared to ten according to LR-NGS.

##### 3.4.2.5 Macrolide–Lincosamide–Streptogramin Resistance

MLS resistance was determined based on the presence of *Erm*, *msr*, *lsa*, and *vat* genes. *Erm* genes encode erythromycin 23S rRNA methyltransferase, which confers resistance to macrolide, lincosamide, and streptogramin B. *ErmA* genes were detected in four and eight isolates using SR- and LR-NGS, respectively, while *ErmB* genes were detected in 16 and 17 isolates, respectively. In addition, the *Erm (33)* gene was found in one sample by LR-NGS. The *msrC* gene, which encodes an ABC-F ribosomal protection protein that confers resistance to macrolide and streptogramin B, was detected in four and three isolates by SR- and LR-NGS, respectively; one sample was common to both methods. The *lsaA* gene, which belongs to the ABC-F subfamily and confers resistance to lincosamide and streptogramin A (Sa), and the *vatD* gene (also known as the Sa acetyltransferase) were detected in one sample by LR-NGS. In total, 17 and 18 isolates were shown to be MLS resistant by SR- and LR-NGS, respectively (**Tables 7**, **8**).

**Table 7:** Genes detected by short-read next-generation sequencing that confer antimicrobial resistance (AMR) to macrolide–lincosamide–streptogramin (MLS).

Isolate	AMR genes conferring MLS Resistance					
	*ErmA*	*ErmB*	*Erm(33)*	*msrC*	*lsaA*	*vatD*
EF01	**-**	**+**	**-**	**-**	**-**	**-**
EF02	**-**	**+**	**-**	**-**	**-**	**-**
EF03	**+**	**+**	**-**	**+**	**-**	**-**
EF04	**+**	**+**	**-**	**+**	**-**	**-**
EF05	**-**	**+**	**-**	**-**	**-**	**-**
EF06	**-**	**+**	**-**	**-**	**-**	**-**
EF07*	**-**	**+**	**-**	**-**	**-**	**-**
EF08	**+**	**+**	**-**	**+**	**-**	**-**
EF09	**-**	**+**	**-**	**-**	**-**	**-**
EF10	**-**	**+**	**-**	**-**	**-**	**-**
EF11	**-**	**+**	**-**	**-**	**-**	**-**
EF12	**-**	**+**	**-**	**-**	**-**	**-**
EF13*	**-**	**+**	**-**	**-**	**-**	**-**
EF14	**-**	**+**	**-**	**-**	**-**	**-**
EF15	**-**	**+**	**-**	**-**	**-**	**-**
EF16*	**-**	**-**	**-**	**-**	**-**	**-**
EF17	**+**	**+**	**-**	**+**	**-**	**-**
EF22	**-**	**+**	**-**	**-**	**-**	**-**
EF23†	**-**	**-**	**-**	**-**	**-**	**-**
**Total**	4	17	0	4	0	0
	**17**					

*Enterococcus faecalis.

^†^Enterococcus casseliflavus.

**Table 8:** Genes detected by long-read next-generation sequencing that confer antimicrobial resistance (AMR) to macrolide–lincosamide–streptogramin (MLS).

Isolate	AMR genes conferring MLS Resistance					
	*ErmA*	*ErmB*	*Erm(33)*	*msrC*	*lsaA*	*vatD*
EF01	**+**	**+**	**-**	**-**	**-**	**-**
EF02	**-**	**+**	**-**	**+**	**-**	**-**
EF03	**+**	**+**	**-**	**-**	**-**	**-**
EF04	**+**	**-**	**-**	**-**	**-**	**-**
EF05	**-**	**+**	**-**	**-**	**-**	**-**
EF06	**+**	**+**	**-**	**-**	**-**	**-**
EF07*	**-**	**+**	**-**	**-**	**-**	**-**
EF08	**+**	**+**	**-**	**+**	**-**	**-**
EF09	**+**	**+**	**-**	**+**	**-**	**-**
EF10	**+**	**+**	**-**	**-**	**-**	**-**
EF11	**-**	**+**	**-**	**-**	**-**	**-**
EF12	**-**	**+**	**-**	**-**	**-**	**-**
EF13*	**-**	**+**	**-**	**-**	**-**	**-**
EF14	**-**	**+**	**-**	**-**	**-**	**-**
EF15	**-**	**+**	**-**	**-**	**-**	**-**
EF16*	**-**	**-**	**-**	**-**	**+**	**-**
EF17	**+**	**+**	**+**	**-**	**-**	**-**
EF22	**-**	**+**	**-**	**-**	**-**	**+**
EF23†	**-**	**-**	**-**	**-**	**-**	**-**
**Total**	8	16	1	3	1	1
	**18**					

*Enterococcus faecalis.

^†^Enterococcus casseliflavus.

##### 3.4.2.6 Other Types of AMR

The *SAT-4* gene was identified in five isolates by both SR- and LR-NGS; this gene encodes streptothricin acetyltransferase, which confers resistance to streptothricin. Four additional isolates were shown to harbor *SAT-4* by SR-NGS. Also, several AMR genes encoding multidrug resistant efflux pumps were detected. The most common of these genes was *efmA*, followed by *efrAB* and *emeA* according to both SR- and LR-NGS (data not shown).

## 4 Discussion

Enterococci are one of the most problematic nosocomial pathogens because they usually affect patients already suffering from debilitating diseases. Enterococcal infection typically necessitates prolonged hospitalization, imposes an economic burden, and increases mortality and morbidity (Cosgrove, 2006). Bacterial resistance to commonly used antibiotics is widespread. For example, enterococci are intrinsically resistant to β-lactams like cephalosporins, penicillin, and (in the case of *E. faecium*) ampicillin, as well as clindamycin and low-level aminoglycosides. They also have the capacity to acquire and disseminate genetic determinants of AMR such as vancomycin, erythromycin, and high-level aminoglycosides (Eliopoulos and Gold, 2001; Gagetti et al., 2019). Thus, the World Health Organization has designated VREfm as a priority pathogen to encourage national surveillance programs (Tacconelli et al., 2018).

To prevent the spread of VRE in the hospital setting, the epidemiology of nosocomial infections should be studied, and that requires understanding the genetic relatedness of pathogens. Molecular approaches to the diagnosis and epidemiological analysis of nosocomial infections aid identification of infection sources and distinctive AMR patterns (Singh et al., 2006; Humphreys and Coleman, 2019). We used WGS for epidemiological analysis of VRE in an attempt to establish a standardized workflow. We performed strain typing of VRE isolates using SNP-based methods, along with genetic profiling of genes and mutations conferring AMR.

The popularity of NGS in clinical microbiology has increased dramatically for several reasons. WGS enables high-resolution analysis of entire genome sequences, and the cost is continually falling. Moreover, the availability of graphical user interface-based software for bioinformatic analysis is increasing (Allard et al., 2012; Tan et al., 2018). Although several studies have demonstrated the effectiveness of WGS for bacterial strain typing, including of VRE, they mostly focused on the discriminatory power of strain typing methods used in conjunction with sequencing analysis. For example, one study found that SNP-based strain typing was slightly more discriminatory than whole-genome MLST (Pearce et al., 2018). To our knowledge, few studies have compared different sequencing platforms in the context of the molecular epidemiology of bacteria. Neal-McKinney et al. (2021) compared the efficacy of MiSeq, MinION, and hybrid genome sequencing for the analysis of *Campylobacter jejuni*, but their comparison focused only on the preprocessing of sequencing data and read assemblies.

Despite the clear advantages of WGS-based bacterial epidemiology, some challenges remain. The main priority is to standardize workflows and pipelines, including sequencing methods and bioinformatic analyses, to ensure sensitivity and specificity. Moreover, many variables in the bioinformatic analysis are likely to affect the utility and overall performance of pipelines for QC and data trimming, bioinformatic tools and algorithms, reference databases, and thresholds for positive results. One of them is to select a proper read assembly technique based on the sequencing platform as well as downstream analysis to proceed. Herein, we chose to perform variant detection following reference-based assembly to assess genetic relatedness, and *de novo* assembly to enable the identification of important loci in the accessory genome like AMR genes (Scheunert et al., 2020);. In the case of a genome assembly using error-prone long reads, post-sequencing correction can increase the accuracy of the sequences. The correction algorithms include a hybrid method, which in addition uses short reads for assembly and polishing, and the long-read-only method such as consensus polishing *via* POA graphs (Rang et al., 2018; Lee et al., 2021). Thus, pipelines should be validated based on real-world data before being applied in clinical laboratories.

Our in-house bacterial WGS pipelines take account of the variation among sequencing platforms. Essentially, our pipelines constitute three-step analyses of SR- and LR-NGS data for the purposes of strain typing and AMR profiling. The three steps are as follows: read preprocessing and assembly (primary analysis), variant calling (secondary analysis), and strain typing and AMR detection (tertiary analysis). In this study, the steps were evaluated using clinical VRE isolates, and the results were compared between SR- and LR-NGS. For strain typing, we performed reference-based assemblies to analyze the SNPs of VRE isolates and constructed SNP-based phylogenies using a maximum-likelihood algorithm. SR-NGS detected significantly more SNPs than LR-NGS (**Figure 2**); this was attributed to its higher resolution for detecting nucleotide substitutions of bacterial sequences, which led to differences in the phylogenetic trees generated by SR- and LR-NGS. Branch length reflects the substitution rate, and the phylogenetic tree generated by SR-NGS generally had longer branches than that of LR-NGS. For AMR profiling, reads were subjected to *de novo* assemblies to determine genetic determinants of AMR in each isolate, and we analyzed the concordance between genotypic and phenotypic resistance. In particular, we examined resistance to daptomycin, which is a last-resort antibiotic for VRE infection. A previous study proposed that mutations in the *liaF* and *gdpD* genes were responsible for daptomycin resistance (Palmer et al., 2011), but these mutations were not detected by the SR- or LR-NGS conducted in this study; every isolate was phenotypically susceptible to daptomycin (data not shown). In general, the concordance between the genotypic and phenotypic resistance of the antibiotics was better for LR-NGS than SR-NGS. We attributed the few discrepancies to the fact that the relationship between AMR genes/mutations and phenotypic resistance is influenced by multiple factors, including genes not directly involved in known drug-resistant machineries but required to express resistance, and an interplay among the mechanisms of AMR also exists (Yeh et al., 2006; Girgis et al., 2009; Suzuki et al., 2014). Moreover, the AMR databases used for NGS analyses are incomplete, because many genes involved in genotypic and phenotypic resistance remain to be discovered.

## Data Availability Statement

The datasets presented in this study can be found in online repositories. The names of the repository/repositories and accession number(s) can be found below: https://www.ncbi.nlm.nih.gov/, PRJNA798443.

## Author Contributions

SO and KP conceived and planned the experiments. HC and SN performed DNA extraction and next-generation sequencing. SO, KP, and HC analyzed the data and interpreted the results. SO performed the statistical analysis and wrote the manuscript. SO, KP, HC, and SN contributed to the final version of the manuscript and approved the submitted version.

## Funding

This work was supported by the research fund No. 2019-ER5402-01 from the Korea Disease Control and Prevention Agency.

## Conflict of Interest

The authors declare that the research was conducted in the absence of any commercial or financial relationships that could be construed as a potential conflict of interest.

## Publisher’s Note

All claims expressed in this article are solely those of the authors and do not necessarily represent those of their affiliated organizations, or those of the publisher, the editors and the reviewers. Any product that may be evaluated in this article, or claim that may be made by its manufacturer, is not guaranteed or endorsed by the publisher.

## Nomenclature

VREfm, vancomycin-resistant *Enterococcus faecium*; SR-NGS, short-read next-generation sequencing; LR-NGS, long-read next-generation sequencing.
