# Supplementary material for: Comparative Analysis of Short- and Long-Read Sequencing of Vancomycin-Resistant Enterococci for Application to Molecular Epidemiology
Source: Front Cell Infect Microbiol. 2022 Apr 6;12:857801. doi: 10.3389/fcimb.2022.857801 (PMC9019564; doi:10.3389/fcimb.2022.857801)
Supplement: Supplementary file 1 [file Table_1.docx]

Supplementary Material.

**Supplementary Table 1.** Species identification and AMR of the representative antibiotics determined by biochemical tests and predicted by SR- and LR-NGS

| **Isolate** | **Origin** | **Species identification** | |  | **Vancomycin resistance†** | | |  | **Teicoplanin resistance†** | | | |  | **Aminoglycoside resistance†** | | | | | |  | **Tetracycline resistance†** | | |
| --- | --- | --- | --- | --- | --- | --- | --- | --- | --- | --- | --- | --- | --- | --- | --- | --- | --- | --- | --- | --- | --- | --- | --- |
|  |  | **Biochemical ID** | **NGS** |  | **MIC** | **SR-NGS** | **LR-NGS** |  | **MIC** | | **SR-NGS** | **LR-NGS** |  | **MIC*** | |  | **SR-NGS** | | **LR-NGS** |  | **MIC** | **SR-NGS** | **LR-NGS** |
|  |  |  |  |  |  |  |  |  |  |  |  |  |  | **Gentamicin** | **Streptomycin** |  |  |  |  |  |  |  |  |
| EF01 | Clinical | *E. faecium* | *E. faecium* |  | R | R | R |  | I | R | | R |  | S | S |  | R | R | |  | S | S | S |
| EF02 | Clinical | *E. faecium* | *E. faecium* |  | R | R | R |  | I | R | | R |  | R | S |  | S | R | |  | S | S | S |
| EF03 | Clinical | *E. faecium* | *E. faecium* |  | R | R | R |  | I | R | | R |  | S | S |  | R | R | |  | S | R | S |
| EF04 | Clinical | *E. faecium* | *E. faecium* |  | R | R | R |  | R | R | | R |  | S | S |  | R | R | |  | S | R | S |
| EF05 | Clinical | *E. faecium* | *E. faecium* |  | R | R | R |  | R | R | | R |  | S | S |  | R | S | |  | S | S | S |
| EF06 | Clinical | *E. faecium* | *E. faecium* |  | R | R | R |  | R | R | | R |  | S | S |  | R | R | |  | S | S | S |
| EF07 | Clinical | *E. faecium* | *E. faealis* |  | R | S | R |  | R | S | | R |  | l | S |  | R | R | |  | R | S | S |
| EF08 | Clinical | *E. faecium* | *E. faecium* |  | R | R | R |  | I | R | | R |  | S | S |  | R | R | |  | S | R | S |
| EF09 | Clinical | *E. faecium* | *E. faecium* |  | R | R | R |  | S | R | | R |  | R | S |  | R | R | |  | S | S | S |
| EF10 | Clinical | *E. faecium* | *E. faecium* |  | R | R | R |  | I | R | | R |  | S | S |  | S | R | |  | S | S | S |
| EF11 | Clinical | *E. faecium* | *E. faecium* |  | R | R | R |  | S | S | | S |  | R | S |  | R | R | |  | S | S | S |
| EF12 | CAP | *E. faecium* | *E. faecium* |  | R | S | R |  | R | S | | R |  | S | R |  | S | R | |  | S | S | S |
| EF13 | CAP | *E. faecium* | *E. faealis* |  | R | R | R |  | R | R | | R |  | R | S |  | R | R | |  | R | S | S |
| EF14 | CAP | *E. faecium* | *E. faecium* |  | R | R | R |  | R | R | | R |  | S | R |  | R | R | |  | S | S | S |
| EF15 | CAP | *E. faecium* | *E. faecium* |  | R | R | R |  | R | R | | R |  | R | R |  | S | R | |  | R | S | S |
| EF16 | Clinical | *E. faealis* | *E. faealis* |  | S | S | S |  | S | S | | S |  | S | S |  | S | S | |  | R | R | S |
| EF17 | Clinical | *E. faecium* | *E. faecium* |  | S | S | S |  | S | S | | S |  | S | S |  | R | R | |  | S | S | S |
| EF22 | CAP | *E. faecium* | *E. faecium* |  | R | R | R |  | R | R | | R |  | R | S |  | R | R | |  | R | S | R |
| EF23 | CAP | *E. faecium* | *E. casseliflavus* |  | R | R | R |  | S | S | | S |  | S | S |  | S | S | |  | S | S | S |

Abbreviations: ID, identification; AMR, antimicrobial resistance; CAP, College of American Pathologist; MIC, minimal inhibitory concentration; NGS, next-generation sequencing; R, resistant; I, intermediate; S, susceptible; SR-NGS, short-read next-generation sequencing; LR-NGS, long-read next-generation sequencing.

*Biochemical aminoglycoside resistance was only tested for high-level gentamicin and streptomycin.

†The AMR of the representative antibiotics determined by biochemical MIC tests and NGS showed few discrepancies since the relationship between AMR genes/mutations and phenotypic resistance is influenced by multiple factors and the AMR databases used for NGS analyses are incomplete.
